# Multiple regulatory mechanisms, functions and therapeutic potential of chaperone-mediated autophagy

**DOI:** 10.7150/thno.107761

**Published:** 2025-02-03

**Authors:** Yuhan Wang, Jingwei Liu, Hao Wang, Pengcheng Jiang, Liangzi Cao, Songming Lu, Siyi Zhang, Ruohan Yang, Hao Feng, Liu Cao, Xiaoyu Song

**Affiliations:** 1 The College of Basic Medical Science, Health Sciences Institute, China Medical University, Shenyang, Liaoning Province 110122, China.; 2 Key Laboratory of Cell Biology of Ministry of Public Health, Key Laboratory of Medical Cell Biology of Ministry of Education, Key Laboratory of Precision Diagnosis and Treatment of Gastrointestinal Tumors of Ministry of Education, Liaoning Province Collaborative Innovation Center of Aging Related Disease Diagnosis and Treatment and Prevention, China Medical University, Shenyang, Liaoning Province 110122, China.; 3 Department of Gastroenterology, Shengjing Hospital of China Medical University, Shenyang, China.; 4 Department of Ophthalmology, The First Affiliated Hospital of China Medical University, Shenyang, Liaoning, 110001, China.

**Keywords:** autophagy, chaperone-mediated autophagy, post-translational modifications, Hsc70, LAMP2A.

## Abstract

Autophagy refers to the proteolytic degradation of cytoplasmic components by lysosomes, and includes three defined types: macroautophagy, chaperone-mediated autophagy (CMA), and microautophagy. Although the regulatory pathways of macroautophagy are well defined, how CMA is accurately regulated remains less understood. In recent years, emerging evidence has suggested that chaperone-mediated autophagy is regulated by multiple mechanisms at nucleic acid and protein levels. In this review, we summarized recent progress on multiple regulatory mechanisms and functions concerning CMA, as well as novel treatments targeting specific regulation sites.

## Introduction

Autophagy refers to the proteolytic degradation of cytoplasmic components by lysosomes, and includes three defined types: macroautophagy, chaperone-mediated autophagy (CMA) and microautophagy 1. Autophagy is critical for the maintenance of homeostasis by eliminating degenerated cellular components such as proteins and organelles 2. Of the three major patterns of autophagy, CMA is the first to be identified as a process that can be selective 3. The mechanism of CMA includes three main components: the substrate to be degraded, a chaperone molecule, and a receptor on the lysosome membrane 4. Chaperone-mediated autophagy specifically degrades proteins with a pentapeptide motif, KFERQ. Such a substrate is recognized by heat shock protein family A (HSP70) member 8 (HSPA8; also known as heat shock cognate protein 70 [Hsc70]) and is subsequently delivered to the lysosomal membrane 4-6. The lysosomal receptor, lysosomal associated membrane protein 2A (LAMP2A), is responsible for transporting the substrate into the lysosomal lumen for degradation 7. Chaperone-mediated autophagy has been reported to regulate a variety of biological processes 4.

Emerging evidence suggests that chaperone-mediated autophagy is regulated by multiple mechanisms. From the perspective of CMA substrates, the RNA around the substrate 8 or changes in the gene of the substrate itself 9 can affect its degradation. In addition, the occurrence of CMA is closely related to the post-translational modifications (PTMs) of substrates. PTMs of proteins are special chemical groups that attach to amino acid side chains by covalent binding, including phosphorylation, acetylation, ubiquitination, methylation, and so on 10, 11. PTMs of CMA substrates could change the recognition and binding between the substrates and CMA component molecules (Hsc70 and LAMP2A). Similarly, the chaperone molecules Hsc70 and receptor LAMP2A are also regulated by nucleic acids and PTMs 12-14

CMA modulates various cellular changes, including cell proliferation 15, cell cycle 16, metabolism 17, immunity 18, aging 19, cell death [20]and so on. Furthermore, targeting CMA could benefit the treatment of cancer 21, neurodegenerative diseases 22, 23, hepatic diseases 24, 25, kidney diseases 26 and diabetic complications 27. In this review, we systematically summarized recent advances of the regulation, the effects on cellular changes and the therapeutic potential of CMA.

## Regulatory mechanisms of chaperone-mediated autophagy

### Substrates of chaperone-mediated autophagy

#### Basic characteristics of CMA substrates

The characteristic of CMA substrates is certain protein with KFERQ motif 28. Actually, it is the properties of the amino acid residues composing the pentapeptide motif that determines whether a substrate can be degraded by CMA 29. PTMs of substrates (like phosphorylation, acetylation) could change the motif composition and promote CMA degradation by producing similar targeting motifs 30. For instance, acetylated K (Lys) can replace Q (Gln) because acetylation enables K to acquire properties similar to Q 30-32.

#### Regulation of nucleic acids on CMA substrates

DNA mutation of substrates and their regulator, as well as substrate interacting RNAs have been suggested to regulate substrates degradation through CMA. V617F mutation of the Janus kinase 2 (JAK2) gene (JAK2^V617F^) reduced the degradation of pyruvate kinase M1 (PKM1) through CMA by blocking Hsc70-PKM1 binding, which leads to STAT3 activation and promotes the production of interleukin-6 33. The LFSINE insertion sequence in a hematopoietic-specific member of the Rho GTPase family (RhoH) promoted the degradation of RhoH through CMA since deletion of this sequence delayed the degradation of RhoH, suggesting that this sequence may be a signal for lysosomal uptake 9. In addition, long noncoding RNA (lncRNA) RNA component of mitochondrial RNA-processing endoribonuclease (RMRP) interacts with Small nuclear ribonucleoprotein polypeptide A' (SNRPA1) and isolates it in the nucleus, which attenuates the contact between SNRPA1 and CMA component molecules and inhibits the CMA degradation of SNRPA1 in the cytoplasm 8.

#### Regulation of phosphorylation on CMA substrates

Phosphorylation is involved in almost every biochemical process, including cell division, regulation of gene expression, and signal transduction 34. Phosphorylation of CMA substrates can affect the tendency of substrates to be degraded, which can be enhanced or weakened. ULK1 phosphorylation mimic variants (ULK1 S423D) bound more CMA component molecules than those in wild-type cells, which suggests that ULK1 phosphorylation increased its interaction with LAMP2A or Hsc70 35. Microrchidia family CW-type zinc finger 2 (MORC2) is phosphorylated at T717 and T733 by cyclin-dependent kinase 1 (CDK1), which is activated by paclitaxel and vincristine. The phosphorylation at T717 and T733 enhances the combination of MORC2 with Hsc70 and LAMP2A 36. However, in another situation, protein kinase cAMP-activated catalytic subunit alpha (PRKACA), activated by G protein-coupled estrogen receptor 1 (GPER1), phosphorylates MORC2 at T582, thus triggering a reduction in the interaction between MORC2 and CMA component molecules and protecting MORC2 from lysosomal degradation through CMA 37. Phosphorylation of BCL2 binding component 3 (BBC3) at Ser10, mediated by IKKβ (inhibitor of kappa light polypeptide gene enhancer in B-cells, kinase β), prevents its degradation by CMA 38. The accumulation of α-synuclein (α-syn) has been implicated in the pathogenesis of Parkinson's disease (PD). S129E mutant α-syn (mimic phosphorylated α-syn) failed to translocate across the lysosomal membrane compared to the wild-type α-syn, indicating that phosphorylated α-syn could not degrade through CMA 39. Phosphoprotein Enriched in Astrocytes of 15 kDa, a death effector domain family member (PED) is identified as a substrate for CMA. The major phosphorylation of PED at Ser104 and Ser116 attenuates the combination of PED and Hsc70, thus restraining PED degradation through CMA 40. Proviral Insertion in the murine lymphomas 2 (PIM2) oncogene phosphorylates hexokinase-II (HK2) at T473. Through the knockdown of Hsc70 and blockade of CMA, PIM2-mediated T473 phosphorylation of HK2 was proved to be responsible for the stability of HK2 41.

#### Regulation of acetylation on CMA substrates

Acetylation is a biochemical reaction that transfers acetyl groups from acetyl coenzyme A (acetyl-CoA) to specific proteins, thereby regulating the effects and functional diversity of certain proteins 42. In the acetylation reaction, acetyltransferase and deacetylase are responsible for the modification and removal of acetyl groups to substrate proteins 42. Acetylation of 17β-hydroxysteroid dehydrogenase type 4 (HSD17B4) at K669 by dihydroxytestosterone enhances its degradation through CMA, thus promoting its degradation in prostate cancer (PCa) cells 43. Estrone (E1) also enhances K669 acetylation of HSD17B4 and promoted its degradation 44. M2 isoform of pyruvate kinase (PKM2) acetylation at K305 promoted its binding to Hsc70 and enhanced its degradation through CMA, which ultimately promoted tumor growth 31. The stress response protein regulated in development and DNA damage response 1 (REDD1) are associated with complications of recognition by Hsc70 45. Generally, the above results indicate that substrate acetylation can promote the occurrence of CMA or is a prerequisite for CMA.

The acetylation of the substrate might also exert an inhibitory effect on CMA. Tau is a microtubule-binding protein in cells and its abnormal deposition is associated with the onset of many diseases, collectively referred to as tauopathies 46. Tau acetylation induced by the loss of TSC1 prevents its clearance through CMA, thereby increasing the risk of tauopathy 47. However, in another study, an increase in acetylated tau did reduce CMA, but by affecting substrate translocation within the lysosomes, rather than interfering with substrate binding to the surface of the lysosomes 48. Mammalian hepatitis B X-interacting protein (HBXIP), highly expressed in breast cancer tissues, is involved in cellular proliferation and invasion as an oncoprotein 49. HBXIP promotes K277 acetylation of homeobox B13 (HOXB13, a transcription factor) and prevents its degradation by CMA, which eventually leads to drug resistance in breast cancer 50.

Not only does acetylation itself affect CMA, but the enzymes (like deacetylase) involved in acetylation also regulate CMA. The expression of silent information regulator 3 (SIRT3) in hepatocytes activates AMP-activated protein kinase (AMPK) and promotes the formation of a LAMP2A-Hsc70-PLN2 complex, which, in turn, promotes the occurrence of CMA 51. Besides, the activity of CMA is affected by histone deacetylase 6 (HDAC6)-mediated HSP90 deacetylation 52. In a HeLa cell line with HDAC10 knockdown, the substrate Glyceraldehyde-3-phosphate dehydrogenase (GAPDH) was found to be more efficiently degraded by CMA, indicating that knockdown of HDAC10 activated CMA and accelerated substrate degradation 53.

#### Regulation of other PTMs on CMA substrates

In addition to phosphorylation and acetylation, ubiquitination, palmitoylation, SUMOylation and glycosylation are also involved in the regulation of CMA substrates. Ubiquitination is a type of reversible PTMs using ubiquitin as a substrate, and it is catalyzed by a series of enzymes called E1 ubiquitin-activating enzymes, E2 ubiquitin-conjugating enzymes, and E3 ubiquitin ligases 54-56. K63 ubiquitination of hypoxia inducible factor 1, α subunit (HIF1A) is crucial for its degradation through CMA. The inhibition of this type of ubiquitination hindered the degradation of HIF1A by CMA 57. Palmitoylation is a reversible special case among many irreversible lipid modification reactions, which modifies a sixteen-carbon saturated fatty acid (palmitate) to the cysteine residue of the protein in the form of thioesterification. Palmitoylation of most proteins is carried out by palmitoyl S-acyltransferases, which are part of the zinc finger DHHC-type containing (ZDHHC) family 58, 59. ZDHHC12 catalyzes palmitoylation of nucleotide-binding oligomerization domain, leucine-rich repeat and pyrin-domain-containing 3 (NLRP3) at C844. This palmitoylation enhances its recognition by Hsc70, thus promoting its degradation through CMA 60. SUMOylation is a type of PTMs using small ubiquitin-like modifier (SUMO) as the substrate, which usually modifies lysine residues in protein 61. Like ubiquitin, a SUMO protein also undergoes a cascade reaction *in vivo* to modify the target protein, including the action of enzymes E1, E2, and E3 62, 63. SUMOylation of stimulator of interferon genes (STING) by the E3 ubiquitin ligase TRIM38 at K337 is located in the attachment of the QEVLR motif (amino acids 326-330) in STING, thus leading to a masking effect of modified SUMO molecules on the motif and inhibition of the interaction of Hsc70 with STING 64. Glycosylation refers to the selective modification of amino acid residues of substrate proteins by monosaccharides or glycans, including N-glycosylation, O-glycosylation, and C-glycosylation according to the different covalent bonds between proteins and glycogroups 65. After human papillomavirus (HPV) infection, ULK1 undergoes an O-linked β-N-acetylglucosamine (O-GlcNAc) modification. Since the Ser423 phosphorylation of ULK1 is known to promote the CMA of ULK1 35, it was demonstrated that the O-GlcNAcylation of ULK1 at Ser409 and Ser410 antagonizes its phosphorylation at Ser423 and thus attenuated its degradation via CMA 66.

### Chaperone molecules of chaperone-mediated autophagy

#### Introduction of chaperone molecules

Hsc70 recognizes proteins with KFERQ-like motif and binds with the protein, thus targeting the substrate for lysosomal degradation 5. So far, several co-chaperones have been found to help localize substrate proteins to lysosomes in an Hsc70-dependent manner, including HSP40 (also called as Dnajb1), carboxyl terminus of Hsc70-interacting protein (CHIP) and HSP70-HSP90 organizing protein (HOP), but Hsc70 is the only chaperone found to directly recognize the KFERQ-like motif 6, 67. After Hsc70 recognizes the substrate and binds to it, the HSC70-substrate complex binds to LAMP2A located on the lysosomal membrane 68, 69. Hsc70 then initiates the substrate unfolding with the assistance of several co-chaperones in preparation for the internalization of the substrate into lysosomes 6. After the above processes, Hsc70 dissociates from LAMP2A and is ready to recognize and bind new substrates for further CMA cycle 68.

#### Regulation of chaperone molecules

At present, few studies focus on the regulation of nucleic acids on Hsc70. A mutation or deficiency of PARK7/DJ-1 gene regulates CMA through changing the level of Hsc70. The lack of DJ-1 down-regulates the level of Hsc70 and leads to the aggregation of the substrate α-syn, rather than affecting the number of lysosomes in cells 12.

At PTMs levels, Metformin can activate TAK1-IKKα/β signaling, leading to the phosphorylation of Hsc70 at Ser85 and activation of CMA 13. Generated by the spliceosomal protein, SNRNP70/U1-70K, a P140 peptide is a 21-mer linear peptide (sequence 131-151) that has a phosphorylation site at S140. The P140 peptide inhibits CMA by interfering with the binding of lumenal Hsc70 to substrates 70. SIRT1 mediated deacetylation and deubiquitination of DnaJ heat shock protein family member B1 (Dnajb1), which promotes its binding to Hsc70 and the activity of CMA^71^. CHIP, as a molecular chaperone, promotes the degradation of mutant p53 through lysosomes. This is carried out through K63 ubiquitination, but the specific form of autophagy was not clear and needs to be investigated 72. Methylation is a type of PTMs using the methyl group from S-adenoslyl-L-methionine (SAM) 73. Protein methyltransferases mainly target arginine and lysine residues and are therefore divided into protein arginine methyltransferases (PRMTs) and lysine methyltransferases 74. An effect of a new type of non-histone methyltransferase called methyltransferase-like 21c (Mettl21c) was found to contribute to the stability of Hsc70. Mettl21c trimethylates Hsc70 at lysine 561, thus enhancing its stability and promoting its function in CMA 75. S-nitrosylation is a special PTM mediated by nitric oxide (NO), targeting cysteine residues in substrate proteins. In mammals, NO is produced by nitric oxide synthase (NOS), which mediates the linking of the thiol side chain of cysteine (single or multiple) to nitroso, thus forming a structure called S-nitrosothiol 76, 77. NO-mediated S-nitrosylation of Hsc70 was found to interfere with CMA 78.

### Receptor of chaperone-mediated autophagy

#### Introduction of the receptor

LAMP2A, expressed by the gene LAMP2, is the first identified CMA component molecule located on the lysosome 79, 80. In addition to LAMP2A, this gene also expresses two other splice variants (LAMP2B and LAMP2C), but LAMP2A is the only protein involved in CMA 80, 81. LAMP2A participates in several different steps of CMA, including substrate binding, LAMP2A assembly, substrate translocation, and so on 3. After the formation of the Hsc70-substrate complex, the 12-amino acid tail on the cytoplasmic side of LAMP2A binds to the complex, thus completing the docking of the complex on the lysosome 80, 81. After that, LAMP2A begins to multimerize, forming a 700kDa protein complex that is required for substrate translocation into the interior of the lysosome 68, 82. HSP90 is also involved in LAMP2A assembly and is responsible for stabilizing LAMP2A multimers 68.

#### Regulation of nucleic acids and phosphorylation on LAMP2A

In addition to affecting the level of Hsc70, a mutation or deficiency of PARK7/DJ-1 gene can also regulate CMA by accelerating the degradation of LAMP2A 12. Deficiency of VPS35 gene also accelerates LAMP2A degradation. In VPS35-deficient dopamine neurons, this effect attenuates the CMA of α-syn, causing the abnormal accumulation of α-syn and the development of PD 83.

Some of the transcription factors can bind to the LAMP2 gene, thereby participating in LAMP2A expression to affect CMA. Nuclear factor, erythroid derived 2, like 2 (NFE2L2/NRF2) was found to interact with two sequences in the LAMP2 gene, which can promote the expression of LAMP2A. Therefore, the level of LAMP2A was significantly decreased in NFE2L2-knockout hepatocytes, as well as the activity of CMA 84. Besides, in mouse embryonic stem cells, OCT4 and SOX2 bind with the LAMP2 gene and inhibit CMA 85.

At the RNA level, different kinds of RNA can interfere with LAMP2A expression or interact with LAMP2A, thereby affecting CMA. MicroRNAs, including Homo sapiens (hsa)-miR-224, hsa-miR-320a 86, hsa-miR-373, hsa-miR-379^87^ and hsa-miR-193a-3p^88^ are the main miRNAs that target the LAMP2 gene according to current studies. These miRNAs induced downregulation of CMA and contributed to the accumulation of α-syn in the pathogenesis of PD. In addition, severe acute respiratory syndrome coronavirus 2 (SARS-CoV-2) infection induced the upregulation of LAMP2A. Besides, overexpression of LAMP2A significantly decreased the RNA level of SARS-CoV-2, indicating that LAMP2A has a role in reducing the viral RNA level in SARS-CoV-2-infected cells 89.

The effect of PTMs on LAMP2A is poorly understood, and only phosphorylation has been found to regulate LAMP2A. Endoplasmic reticulum stressors result in activation by a double-stranded RNA-activated protein kinase-like ER kinase, which recruits mitogen-activated protein kinase kinase 4 (MKK4) to lysosomes. The MKK4 can activate p38 mitogen activated protein kinase (MAPK) at lysosomes, which phosphorylates LAMP2A at T211 and T213 directly and activates CMA 14. Beyond that, phosphorylation of upstream molecules also affects LAMP2A. Located on the lysosomal membrane, Akt regulates the phosphorylation of glial fibrillary acidic protein (GFAP) and further affects CMA. Unphosphorylated GFAP promotes the multimerization of LAMP2A by binding the cytosolic tail of LAMP2A, thus accelerating substrate uptake, and facilitates the process of CMA 82, 90. In addition to affecting Hsc70, the above-mentioned P140 peptide also reduces the expression of LAMP2A. Overall, the P140 peptide has a variety of negative effects on the LAMP2A-Hsc70 lysosomal axis, which ultimately leads to the inhibition of CMA 70.

## The effects of chaperone-mediated autophagy on cellular changes

### Cell proliferation

In the studies of esophageal squamous cell carcinoma (ESCC) and non-small cell lung cancer (NSCLC), CMA knockdown inhibited cell proliferation and colony formation, and increased the sensitivity of cancer cells to chemotherapeutic drugs, which provides a new potential target for therapeutic research 15, 91. Using the same approach, it was found that CMA inhibition in colorectal cancer (CRC) cells resulted in a decreased ability of cell migration and invasion, and that overexpression of LAMP2A increased cell viability 92. Some studies found that CMA changes regulate cell proliferation through the action of other molecules. In studies of glioma, additional activation of CMA caused by upregulation of LAMP2A enhanced the proliferation and invasion of cancer cells by promoting the degradation of SMAD3^93^. Besides, the attenuation of CMA caused by LAMP2A knockdown in gastric cancer cells hindered cell proliferation by promoting Rho Family GTPase 3 (RND3/RhoE) accumulation 94. LAMP2A was found to be a potential biomarker for early warning of gastric cancer 94. In addition, co-inhibition of CMA and macroautophagy demonstrated a synergistic effect on the inhibition of proliferation and survival in K-Ras G12V (K-Ras oncogene mutation at G12V) mouse embryonic fibroblasts (MEFs) 95.

It is worth mentioning that downregulation of CMA may also promote cancer cell proliferation due to the lack of degradation of specific molecules. The transcriptional coactivators Yes1 associated transcriptional regulator (YAP1) and interleukin 6 cytokine family signal transducer (IL6ST) were identified as CMA substrates. Knockdown of LAMP2A resulted in increased levels of both the two proteins in hepatocellular carcinoma (HCC) cells, which ultimately promoted the proliferation and migration of HCC cells 96.

### Cell death

Cell death refers to the response of cells to internal and external interference factors or biological signals to inactivate themselves, including apoptosis, necrosis, ferroptosis, cuproptosis, pyroptosis, and so on 97. For example, excessive activation of CMA of hexokinase 2 (HK2, a key enzyme in glucose metabolism) can lead to metabolic disorder of cancer cells and trigger metabolic catastrophe, resulting in the death of cancer cells 98. Furthermore, CMA activation by LAMP2A overexpression reduces hypoxia-induced apoptosis in cardiomyocytes 99, whereas CMA inhibition results in caspase-induced apoptosis occurring in mouse hepatocytes 100.

In fact, so far, the mode of cell death that has been more frequently studied in relation to CMA is ferroptosis. Among them, glutathione peroxidase 4 (GPX4) is a “bridge” connecting CMA and ferroptosis. GPX4 is considered to be an inhibitor of ferroptosis, and its activity is dependent on glutathione 101. In acute kidney injury, Legumain promotes CMA in renal tubular cells, leading to increased degradation of GPX4, thereby triggering ferroptosis of tubular cells 102. CMA inhibition stabilizes the activity of GPX4 and reduces the occurrence of ferroptosis 103. CMA degradation of GPX4 leads to antimony-induced ferroptosis in neurons 20. Conjugated fatty acids (CFAs) were found to promote CMA of GPX4 and target mitochondria for lipid peroxidation and GPX4 degradation, which provides a new perspective for ferroptosis-related cancer therapy research 104. However, in particular, LAMP2A deficiency was found to increase reactive oxygen species (ROS)-induced ferroptosis in retinal pigment epithelial (RPE) cells, and glutathione (GSH) activity was decreased in LAMP2A-deficient RPE cells 105. In addition to GPX4, CMA of Acyl-CoA synthetase long-chain family member 4 (ACSL4) is also associated with ferroptosis. ACSL4 is an enzyme involved in lipid metabolism and is associated with susceptibility of cells to ferroptosis. Melatonin can promote CMA of ACSL4, thereby reducing the occurrence of ferroptosis 106.

### Cell cycle

HIF1A has been identified as a substrate of CMA 16. As mentioned above, K63 ubiquitination is necessary for HIF1A degradation by CMA 57. HIF1A is responsible for the adaptive response of cells to anaerobic environment, and its overexpression can arrest cells in G1 phase 107. Under hypoxic stress, CMA is a negative regulator of HIF1A, and the inhibition of CMA leads to less degradation of HIF1A and cell cycle arrest 108. Moreover, a pair of cyclin-dependent kinases (CDK1 and CDK2) regulate the degradation of HIF1A through CMA, blocking and promoting HIF1A degradation, respectively 107. In addition, HIF1A-related cell cycle arrest is also associated with the expression of CDK inhibitors p21 and p27 109. The relationship between CMA and the cell cycle was also confirmed by the observation that hypoxia-induced CMA and lysosomal biogenesis in cancer cells are part of a negative feedback regulatory loop 16.

In addition to HIF1A, another CMA substrate implicated in cell cycle regulation is checkpoint kinase 1 (Chk1) 110. Just like the negative feedback regulatory mechanism related to HIF1A, after the DNA of cells was destroyed by etoposide, CMA could be timely upregulated and degrade Chk1 to help resume the cell cycle. CMA inhibition led to the accumulation of Chk1, which blocked normal cell cycle progression and impaired cell viability upon DNA damage 110, 111.

### Immunity

The development and maturation of T cells in the human body is quite essential for maintaining the immune function of the body 112. Itchy E3 ubiquitin protein ligase (Itch) and regulator of calcineurin 1 (Rcan-1) are negative regulators of the T-cell receptor (TCR) signaling pathway, and both inhibit the activation of CD4^+^ T cells 113, 114. CMA maintains CD4^+^ T cell activation by degrading Itch and Rcan-1 18. Itch is a type of E3 ubiquitin ligase responsible for ubiquitinating the Bcl10 Immune Signaling Adaptor (Bcl10), which inhibits the promotion of T-cell activation by the downstream NF-κB signaling pathway 113, 115. In addition, the deficiency of Itch leads to the excessive activation of T cells and causes autoimmune diseases 116. Rcan-1 is a type of calcineurin inhibitor that inhibits the Ca^2+^/ calcineurin/ nuclear factor of activated T-lymphocyte (NFAT) signaling pathway, which promotes T cell activation 114, 117. Furthermore, T cell activation is abnormally suppressed in the LAMP2A-deficient model, accompanied by increased level of Itch and Rcan-1, as well as immune dysregulation 18.

CMA is not only related to specific immunity, but also to non-specific immunity (innate immunity). CMA degradation of NLRP3 is associated with the palmitoylation of NLRP3 60. In fact, NLRP3 is a signaling pathway receptor that regulates the innate immune response by affecting the formation of inflammasomes, which eventually leads to pyroptosis 118. STING degradation via CMA has also been implicated in the regulation of innate immunity, and its role in the cell is to respond to nucleic acids present in the cytosol 64. In addition, TANK binding kinase-1(TBK-1), associated with IFN-1 production during cell infection 119, was identified as a substrate of CMA 120. Inhibition of CMA decreased the degradation of TBK-1 and increased the production of IFN-1, thus improving the antiviral ability of cells 121.

### Metabolism

CMA usually affects the metabolic processes by degrading important enzymes in metabolic pathways. As for glucose metabolism, the degradation of HK2 and PKM2, two essential enzymes in glucose metabolism, was mediated by CMA 31, 98. Besides, the capacity of gluconeogenesis and glycogen storage is weakened by CMA inhibition, while cell glycolysis is also enhanced in LAMP2A-knockout mouse hepatocytes 17. The inhibition of CMA causes the accumulation of CMA substrates responsible for gluconeogenesis including GADPH and malate dehydrogenase, which promotes the glycolysis process 17.

In addition to glucose metabolism, CMA is also associated with lipid metabolism 17. Several proteins involved in lipid metabolism, including lipid droplet coat proteins, lipogenic enzymes, lipid carriers, have been identified as CMA substrates 17, 122. CMA promotes lipolysis by degrading lipid droplet coat proteins such as perilipins 2 and 3(PLIN2 and PLIN3) 122. In addition, aberrant lipogenesis and lipolysis processes are found to cause hepatosteatosis in LAMP2A-knockout mouse hepatocytes 17. Furthermore, CMA can also regulate preadipocyte differentiation by degrading MYC and transforming growth factor-β (TGFβ) during adipogenesis 123.

OCT4 and SOX2, as mentioned above, can regulate CMA by affecting LAMP2 expression. In fact, CMA also has a regulatory effect on the differentiation of embryonic stem cells by regulating the glucose metabolism of embryonic stem cells 85, 124.

### Aging

Aging-dependent decrease in CMA activity has been found in a wide variety of cells 19, 125, 126. LAMP2A is a key rate-limiting protein during CMA, which is also a major factor in the aging-dependent reduction of CMA 127. Alternations in the lipid composition of the lysosomal membrane are found to contribute to the changes in LAMP2A stability during aging 128. In mouse models of hepatocytes and T cells, macroautophagy was found to compensate for a portion of reduced function of CMA 17, 18. However, studies in mouse retinal cells found that only CMA could compensate for the loss of macroautophagy, while macroautophagy could not in turn compensate for the deficiency of CMA 126. Therefore, in general, the loss of CMA function accompanied by aging is irreversible and irreplaceable. Genetic intervention targeting LAMP2A can restore the aging-induced functional deficits. Exogenously induced expression of LAMP2A has been shown to enhance the ability of the aging liver to resist adverse factors, and contribute to the overall improvement of an individual's ability to maintain protein homeostasis 125.

CMA is also involved in the maintenance of bone marrow hematopoietic function during aging. Bone marrow hematopoietic function decreases with aging due to the decline in the number of hematopoietic stem cells and the reduction in the ability of cell renewal. The decrease of CMA also affects the hematopoietic function of bone marrow by causing the glucose metabolism disorder of hematopoietic stem cells. Reactivation of CMA in senescent cells can partially restore the hematopoietic function of bone marrow 129.

## Therapeutic potential of chaperone-mediated autophagy

### Therapeutic potential of CMA on cancers

#### Colorectal cancer

It has been suggested that CMA can promote the proliferation, metastasis, and chemotherapy resistance of colorectal cancer (CRC) cells 21. Targeting and regulation of CMA might contribute to the treatment of CRC. Increased LAMP2A expression was found in colon cancer tissues 92. CMA promotes the metastasis and proliferation of colorectal cancer cells during oxaliplatin resistance and under oxidative stress conditions, which is associated with the process of cancer cell glycolysis 92. The Hsc70/caveolin-1 [CAV1]/β-catenin axis was found to participate in the progression of colorectal cancer. Hsc70 promotes the degradation of CAV1 through CMA, which subsequently activates the Wnt/β-catenin pathway and promotes the metastasis of BRAF V600E CRC 130. Fucosyltransferase 8 (FUT8) is highly expressed in cancer cells, which is responsible for mediating the core fucosylation of the key immune checkpoint molecule, CD276 (B7-H3). FDW028, a specific FUT8 inhibitor, shows potent anticancer activity by promoting degradation of B7-H3 through CMA 131.

#### Breast cancer

The inhibition of CMA is a potential target for the improvement of drug resistance of breast cancer. LAMP2A expression was found to increase in breast cancer tissues. Inhibition of LAMP2A induces apoptosis and increases the sensitivity of breast cancer cells to the drug 132. Hepatitis B X-interacting protein (HBXIP) is an oncoprotein that promotes TAM resistance of cancer cells via the inhibition of the degradation of HOXB13 by CMA. Aspirin can overcome tamoxifen (TAM) resistance in estrogen receptor-positive breast cancer through decreasing the overexpression of HBXIP 50. 17β-estradiol (E2), TAM, and fulvestrant (FUL) stabilized MORC2 through the inhibition of CMA. The knockdown of MORC2 prevented cell proliferation induced by E2 and allowed cancer cells to regain sensitivity to TAM and FUL, thus becoming a potential target of treatment 37.

#### Lung cancer

Knockdown of LAMP2A inhibited tumor progression and enhanced the sensitivity of NSCLC cells to cisplatin, suggesting that inhibition of CMA is a potential approach for the treatment of NSCLC 91. Besides, polyphyllin D suppressed the interaction of Hsc70 with LAMP2A and thus disrupted CMA, contributing to the treatment of NSCLC 133. PI3K/mTOR inhibitors enhanced the interaction of G6PD protein (the rate-limiting enzyme of the pentose phosphate pathway) with Hsc70, thereby enhancing its degradation through CMA, leading to an exacerbation of oxidative stress damage. Therefore, PI3K/mTOR inhibitors may serve as a therapeutic approach to attenuate radioresistance in SCLC 134.

#### Other cancers

CMA and programmed cell death-ligand 1 (PD-L1) upregulation were found to occur simultaneously in metastatic melanoma. Self-assembling prionoid (SAP) was a prion-like chemical inducer of proximity that was designed artificially to induce PD-L1 close to Hsc70 via decomposing into phytohaemagglitinin after infiltrating tumor cells, thus enhancing PD-L1 degradation through CMA 135. For papillary thyroid carcinoma (PTC), CMA regulated by the estrogen receptor can increase the expression of stromal cell-derived factor 1 and C-X-C motif chemokine receptor 4 by reducing the expression of peroxisome proliferator-activated receptor γ, thus promoting the proliferation and metastasis of PTC 136. In the treatment of multiple myeloma, upregulation of CMA contributed to tumor resistance to bortezomib and might serve as a target to overcome resistance 137. Tumor protein D52 (TPD52) mediated the activation of CMA and promoted the proliferation and metastasis of prostatic cancer (PCa). Romidepsin, an inhibitor of HDAC2, increases TPD52 acetylation, which attenuates TPD52-mediated CMA activation and prevents the growth of PCa 138. In non-acute myeloid leukemia cells, inhibition of Fms-like tyrosine kinase 3 led to a sensitivity of cancer cells to autophagy inhibition and an excessive activation of CMA. The activation of CMA increased the degradation of HK2, which triggered the metabolic disorder and impaired proliferation of cancer cells 98.

### Therapeutic potential of CMA on neurodegenerative diseases

#### Parkinson's disease

Parkinson's disease is a neurodegenerative disorder with an age-related onset and is characterized by the accumulation of α-syn in the brain. In general, CMA activation can increase the degradation of α-syn, thereby alleviating the symptoms of PD 22. AR7 [7-chloro-3-(4-methylphenyl)-2H-1,4-benzoxazine], an atypical RARA/RARα (retinoic acid receptor α) antagonist 139, can activate LAMP2A transcription and lysosomal activity, thereby inhibiting the accumulation of α-syn oligomers 23. Besides, β-asarone has a therapeutic effect in a rat model of PD induced by 6-hydroxydopamine, preventing brain damage by increasing MEF2D and tyrosine hydroxylase (TH), and decreasing α-syn through a Hsc70/MAPK/MEF2D/beclin-1 pathway 140. Tubastatin A attenuated the toxicity of α-syn by upregulating HSC70 and LAMP2A 141. In addition to the clearance of α-syn, inhibition of the p38-transcription factor EB pathway can promote the degradation of NLRP3 through CMA, thereby suppressing microglia activation, which is also a potential therapeutic approach of PD 142.

#### Alzheimer's disease

The treatment strategy for Alzheimer's disease (AD) is also the activation of CMA, which leads to the degradation of abnormally aggregated proteins to alleviate the disease. Metformin was reported to activate CMA and significantly reduce the level of Aβ plaque in the brains of mice, thus alleviating the phenotype and symptoms of AD 13. Labeling multiple CMA motifs on pathological plaques can promote their recognition by HSC70 so as to protect nerve cells 143. Lactulose and trehalose were found to reduce neuroinflammation by anti-inflammatory and CMA activation. Moreover, lactulose better promoted synaptic expression in mouse models than trehalose and thus could be used as a potential therapeutic agent for AD 144.

### Therapeutic potential of CMA on hepatic and renal diseases

Aiming at the hepatotoxicity caused by lipid accumulation, the combination of N-acetylcysteine (NAC) and baicalin has been reported to stabilize the function of mitochondria in hepatocytes, thereby inhibiting mitochondrial reactive oxygen species-mediated CMA by transcriptional factor A-choline, promoting lipid phagocytosis and alleviating liver toxicity 25. CMA activators improved proximal tubular function in patients with nephrotic cystinosis. The reason is that CMA activation improved the expression of the endocytic receptor megalin on the cell membrane in proximal tubule cells, thereby restoring its normal function 26.

### Therapeutic potential of CMA on diabetic complications

Glucose toxicity caused by hyperglycemia is the main cause of diabetes-related complications. The role of lmp-2 (LAMP2A homolog) was studied in Caenorhabditis elegans. The inhibition of lmp-2 can prevent decreased proteasome activity caused by glucose, thereby preventing glucose toxicity 145. In diabetic retinopathy (DR), CMA activator QX77 (a derivative of AR7, an RARα antagonist) was found to prevent early DR by promoting the degradation of ACSL4 protein through CMA and preventing the abnormal accumulation that led to the generation of harmful lipid substances 27.

### Therapeutic potential of CMA on microbial infectious diseases

Infection with Salmonella enterica serovar Typhimurium (S. Typhimurium) leads to the death of macrophages. This process is resulted by impairing the degradation of tripartite motif 21 (TRIM21) through CMA by this bacterium and elevated TRIM21 levels eventually led to the death of macrophages. This result links CMA to bacterial infections, suggesting that CMA modulation of bacterial infectious diseases may become a novel therapeutic approach 146. For Group B Coxsackievirus (CVB) infections, anisomycin was reported to inhibit the replication of CVB by promoting the degradation of eukaryotic translation elongation factor 1 alpha 1 (eEF1A1) through CMA, thus becoming a candidate for the treatment of CVB infection 147.

### Therapeutic potential of CMA on other diseases

CMA is closely implicated in the immunosuppression of mesenchymal stromal cells induced by inflammatory cytokines, that is, the inhibition of CMA is a key factor in immunosuppression 148. Besides, pinacidil attenuates myocardial ischemia-reperfusion injury by inhibiting the degradation of calreticulin through CMA 149. For status epilepticus, CMA activity was reported to be upregulated in a rat model and antioxidants such as vitamin E would partially inhibit CMA, thus potentially relieving status epilepticus 150.

## Future perspectives

Recent studies revealed various regulatory mechanisms of CMA as well as the effects of CMA on cellular changes. This review summarized research advances on the regulatory mechanisms of CMA from the perspectives of substrates, chaperone molecule and receptor, and discussed the research and development frontiers of effects on cellular change and therapeutic potential of CMA.

CMA substrates are mainly regulated by PTMs, including phosphorylation, acetylation, ubiquitination and palmitoylation. CMA substrates are characterized by a KFERQ-like motif, which allows the protein to be recognized by Hsc70 for CMA processing. Certain PTMs of the motif can modify the remaining residues to possess properties similar to those in KFERQ, and thus can also be recognized by chaperone molecules. In addition, the modification of residues around the motifs by PTMs can also cause the motifs to be buried or exposed, thereby affecting the recognition of substrates by chaperones. However, little is known on the specific mechanisms of how these PTMs affect the recognition and degradation of CMA component molecules toward substrates. At present, PTMs of Hsc70 phosphorylation, methylation and S-nitrosylation have been studied. Other types of PTMs of Hsc70 might also contribute to the regulation of CMA process. Most researches focused on the regulation of nucleic acids such as miRNA on LAMP2A rather than investigation of PTMs of LAMP2A.

The effects of CMA on cellular changes mainly focused on cell proliferation, cell death, cell cycle, immunity, metabolism, and aging. Other cellular processes such as cell differentiation and cell migration require future investigations. Although most research found that CMA promoted cancer cells proliferation, the relationship of CMA with cell proliferation still need further confirmation as some study came up with contradictory results. Studies on different types of cell death other than ferroptosis might find novel involvement of CMA in cell death regulation. CMA has been reported to maintain normal cell cycle by degradation of relevant proteins. As for immune regulation, CMA could activate CD4^+^ T cells for specific immune reactions as well as innate immune reactions. The glucose and lipid metabolism processes of cells are related to CMA, but the relationship between other metabolic processes such as nucleic acid or amino acid metabolism and CMA still needs further research. During the aging process, the weakening of CMA activity is the main manifestation, and the activation of CMA enhances the ability of aging cells to maintain protein homeostasis.

Investigations on therapeutic potential of CMA mainly focused on malignant tumors, neurodegenerative diseases, liver and kidney diseases. The relationship between CMA and colorectal cancer, breast cancer, lung cancer has been widely studied. CMA can promote the occurrence of cancer, and inhibiting CMA can benefit cancer treatment. In the research of breast cancer, it is only clear that CMA promotes drug resistance of cancer cells, but whether CMA is related to the occurrence of breast cancer needs further research. In addition, whether CMA is related to gastric cancer, pancreatic cancer, liver cancer and other cancers remains to be studied. The neurodegenerative diseases related to CMA mainly include PD and AD. Activation of CMA promotes the degradation of abnormal proteins in neurons, which benefits the treatment of these diseases. Further research is needed to determine whether CMA activation is beneficial for other neurodegenerative diseases such as Huntington's disease and amyotrophic lateral sclerosis. In the complications of diabetes, CMA activation alleviates early diabetes retinopathy, but the relationship between CMA and other complications such as diabetes nephropathy, atherosclerosis remains largely unknown. The advantage of CMA-targeted drugs mainly includes the accurate degradation/stabilization of certain protein for treatment of diseases. While the limitation of CMA-targeted drugs is the possible adverse effect on other systems caused by the significant change of CMA substrate. The diseases such as neurodegenerative diseases and aging might become potential therapeutic objects of CMA-targeted drugs in the future because of the identified relationship and mechanism.

Overall, CMA is regulated at multiple levels of nucleic acid and post-translational modifications which contribute to various cellular processes. Increasing progresses have been made on therapeutic potential of CMA, which may become promising approaches for the effective treatment of multiple diseases.

## Funding

This work was supported by the key project of the National Natural Science Foundation (82030091), the key project of LiaoNing Science Foundation (2022JH6/100100037, 2022JH2/20200034, 2021JH2/10300023), and the National Natural Science Foundation (82102740).

## Figures and Tables

**Figure 1:**
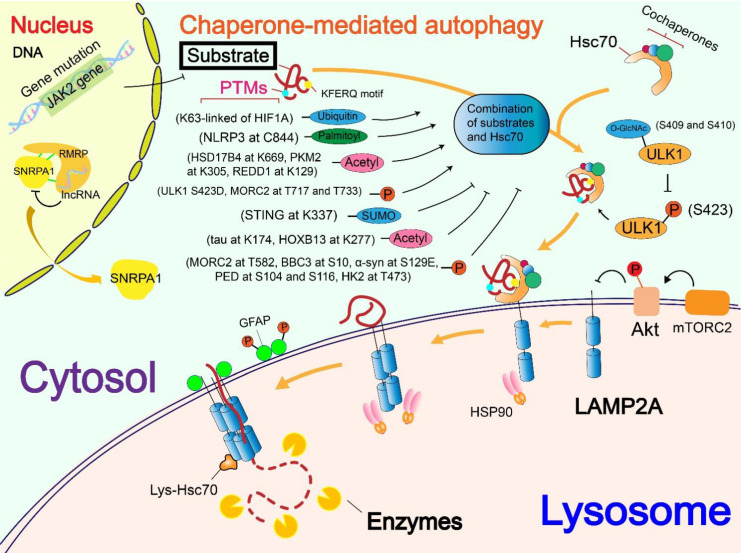
** Regulatory mechanisms of CMA substrates.** Regulation of CMA substrates is divided into regulation of nucleic acids and regulation of the level of post-translational modifications (PTMs). Regulation of nucleic acids includes gene mutations that affect the binding of substrate to Hsc70 (JAK2 gene) and the interaction with substrate to prevent the substrate from entering the cytosol (RMRP). The effects of PTMs levels include phosphorylation, acetylation, ubiquitination, palmitoylation and SUMOylation which modify the substrates and affect the binding of the substrate to Hsc70. In addition, different PTMs of the substrate can interfere with each other, such as the glycosylation modification of ULK1 inhibits the promotion effect of its own phosphorylation on the CMA process.

**Figure 2:**
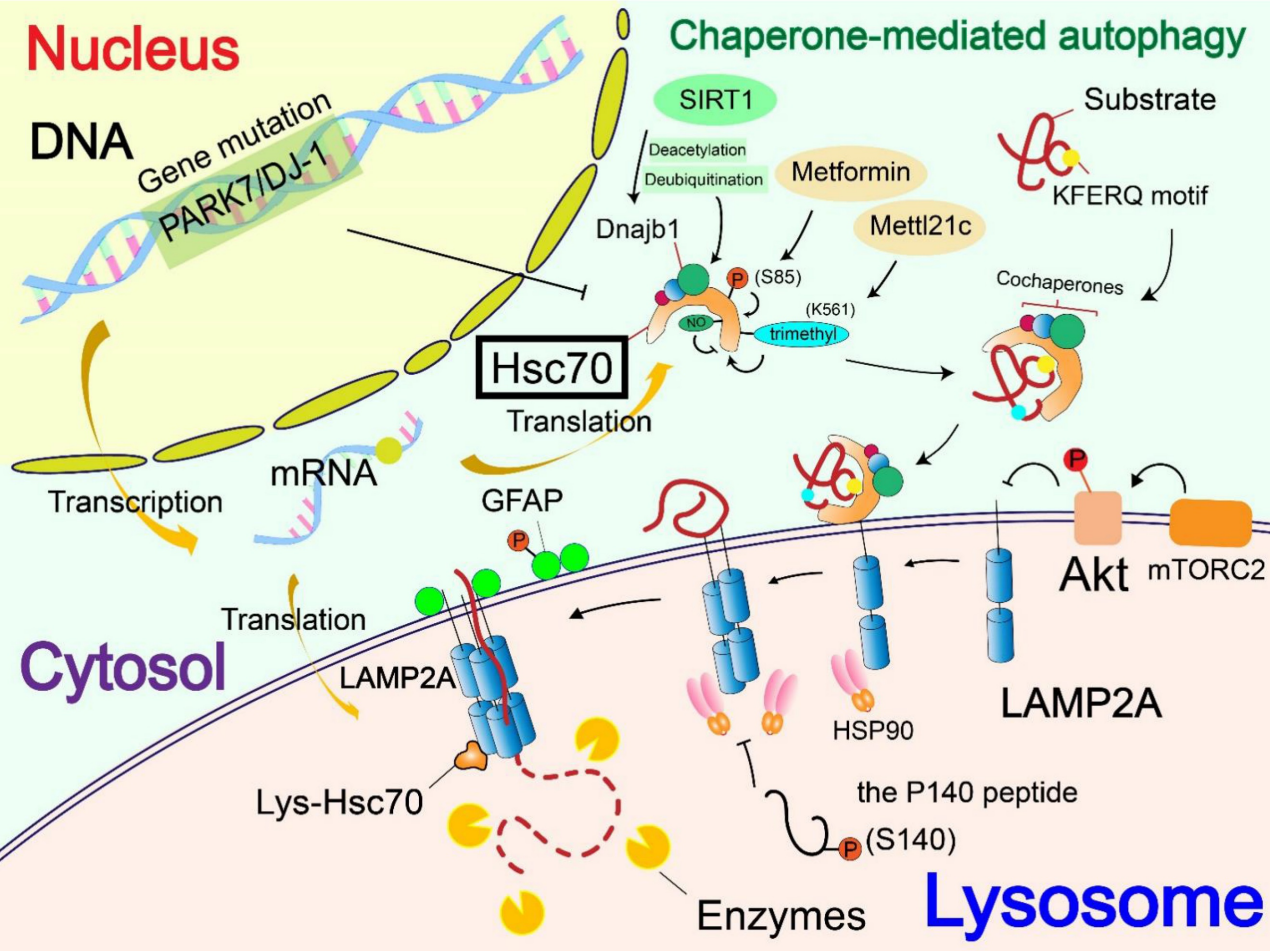
** Regulatory mechanisms of chaperone (Hsc70).** The regulation of Hsc70 is divided into the level of gene mutations and the regulation of Hsc70 by its own PTMs. The mutation of PARK7/DJ-1 gene down-regulates the level of Hsc70, thereby affecting the occurrence of CMA. Phosphorylation and methylation of Hsc70 activate Hsc70, whereas S-nitrosylation inhibits Hsc70 activity. Besides, deacetylase SIRT1 mediated deacetylation and deubiquitination of the cochaperone Dnajb1, thus enhancing its interaction with Hsc70 and regulating CMA.

**Figure 3:**
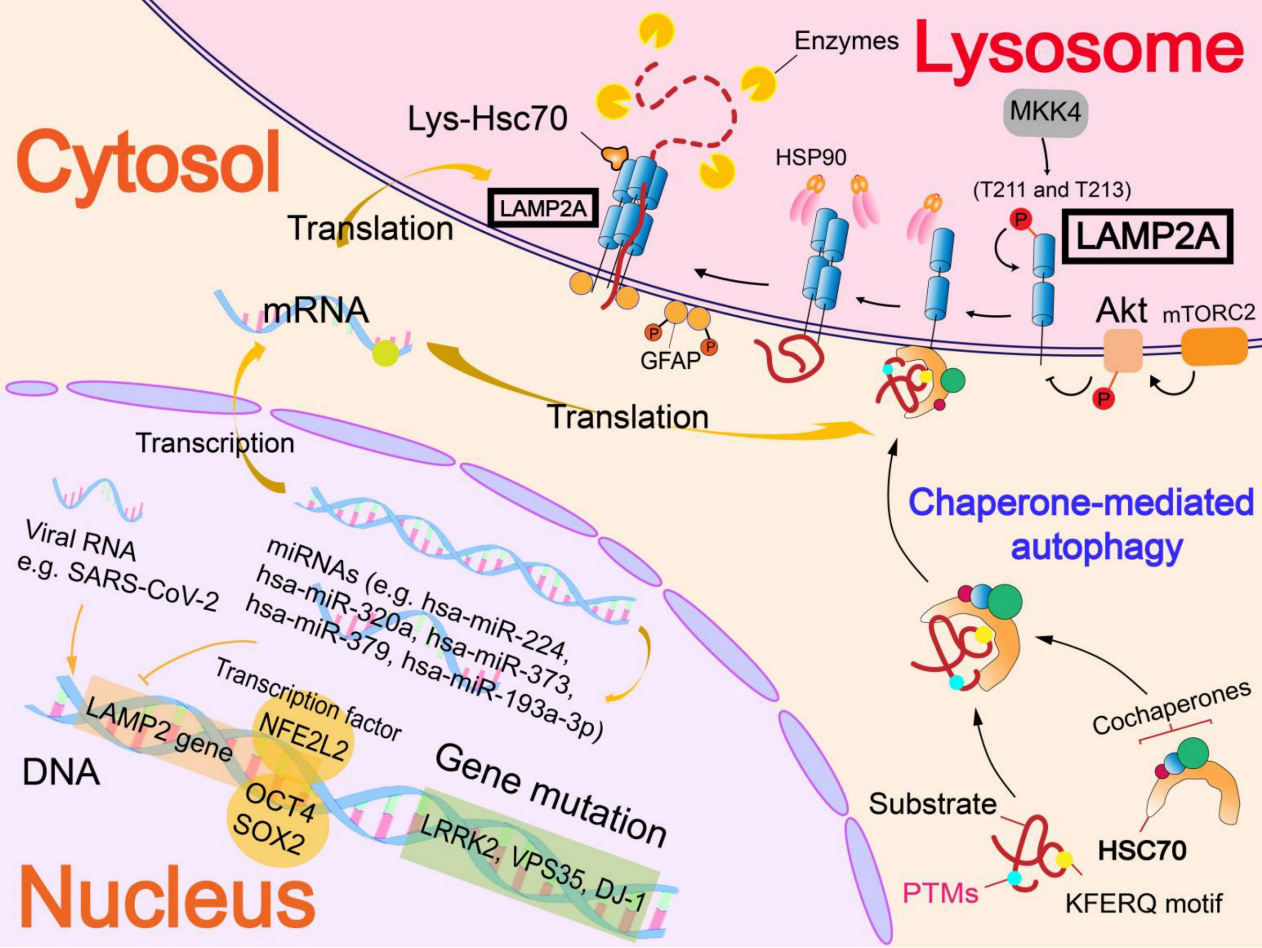
** Regulatory mechanisms of receptor (LAMP2A).** The regulation of LAMP2A mainly focuses on the level of nucleic acids. Gene mutations can accelerate LAMP2A degradation (PARK7/DJ-1 gene and VPS35 gene) or affect lysosomal function (GBA1 gene and LRRK2 gene). Transcription factors interact with LAMP2 gene to up-regulate or down-regulate the level of LAMP2A. MiRNA and viral RNA also interact with the LAMP2 gene to regulate LAMP2A expression. In addition, phosphorylation of LAMP2A can activate LAMP2A to promote CMA.

**Figure 4:**
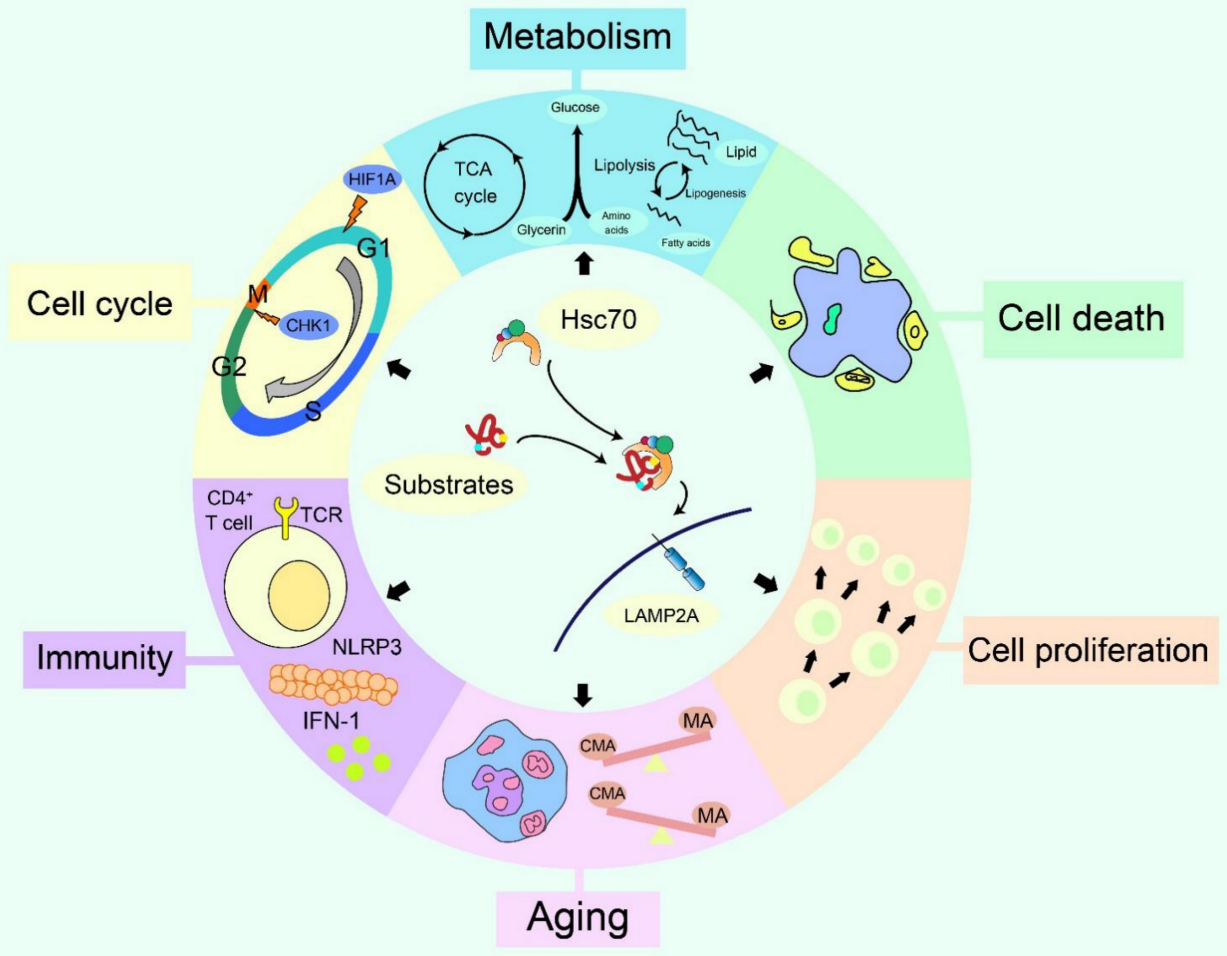
** The effects of CMA on cellular changes.** The cellular changes generated by CMA have six aspects, including cell proliferation, cell death, cell cycle, immunity, metabolism and aging. Up-regulation or down-regulation of CMA can accelerate or slow cell proliferation and death. The relationship between CMA and the cell cycle is mainly due to two CMA substrates that regulate the cell cycle, HIF1A and CHK1. CMA regulates the activation of CD4^+^ T cells and is associated with several processes of innate immunity. In terms of metabolism, CMA interferes with glucose and lipid metabolism and regulates the pluripotency of embryonic stem cells. During aging, a compensatory reaction between CMA and macroautophagy exists to a certain extent, and CMA is also related to the maintenance of bone marrow hematopoietic function during senescence process.

**Table 1:** Targets and sites of regulatory mechanism on CMA substrates.

Substrate	Regulatory mechanism		Regulatory target	Site	Effect on CMA
PKM1	Nucleic acids	V617F mutation of JAK2 gene	Hsc70		inhibited
RhoH		LFSINE insertion sequence	RhoH		enhanced
SNRPA1		lncRNA RMRP	SNRPA1		inhibited
ULK1	Post-translational modifications of proteins	Phosphorylation	ULK1	S423D	enhanced
MORC2			MORC2	T717 and T733	enhanced
MORC2			MORC2	T582	inhibited
BBC3			BBC3	S10	inhibited
α-syn			α-syn	S129E	inhibited
PED			PED	S104 and S116	inhibited
HK2			HK2	T473	inhibited
HSD17B4		Acetylation	HSD17B4	K669	enhanced
PKM2			PKM2	K305	enhanced
REDD1			REDD1	K129	enhanced
tau			tau	K174	inhibited
HOXB13			HOXB13	K277	inhibited
HIF1A		Ubiquitination	HIF1A	K63 linked	enhanced
NLRP3		Palmitoylation	NLRP3	C844	inhibited
STING		SUMOylation	STING	K337	inhibited
ULK1		Glycosylation	ULK1	S409 and S410	inhibited

**Table 2:** Targets and sites of regulatory mechanism on Hsc70.

Regulatory mechanism		Target	Site	Effect on Hsc70	Effect on CMA
Gene mutation	PARK7/DJ-1 gene	Hsc70		Down-regulated	inhibited
Phosphorylation	Metformin	Hsc70	S85	Activated	enhanced
	P140 peptide	Hsc70-HSP90AA1 complex		Interfered	inhibited
Ubiquitination		Dnajb1		Binding of Dnajb1 to Hsc70	enhanced
Deacetylation	SIRT1	Dnajb1			enhanced
Deubiquitination	SIRT1	Dnajb1			enhanced
Methylation	Mettl21c	Hsc70	K561 (trimethylation)	Activated	enhanced
S-nitrosylation	NO	Hsc70		Interfered	inhibited

**Table 3:** Targets and effects of regulatory mechanism on LAMP2A.

Regulatory mechanism		Target	Effect on LAMP2A	Effect on CMA
Gene mutation	PARK7/DJ-1 gene	LAMP2A	Accelerating degradation	inhibited
	VPS35 gene	LAMP2A	Accelerating degradation	inhibited
	GBA1 gene	Lysosome	Causing dysfunction	inhibited
	LRRK2 gene	Lysosome	Causing dysfunction	inhibited
Transcription factors	NFE2L2/NRF2	LAMP2 gene	Up-regulated	enhanced
	OCT4 and SOX2	LAMP2 gene	Down-regulated	inhibited
MiRNA	hsa-miR-224	LAMP2 gene	Down-regulated	inhibited
	hsa-miR-320a	LAMP2 gene	Down-regulated	inhibited
	hsa-miR-373	LAMP2 gene	Down-regulated	inhibited
	hsa-miR-379	LAMP2 gene	Down-regulated	inhibited
	hsa-miR-193a-3p	LAMP2 gene	Down-regulated	inhibited
Viral RNA	SARS-CoV-2	LAMP2A	Up-regulated	enhanced
Phosphorylation	MKK4	LAMP2A at T211 and T213	Activated	enhanced
	Akt	GFAP	Inactivated	inhibited

**Table 4:** Therapeutic molecules targeting chaperone-mediated autophagy.

Therapeutic molecule(s)	Targets	Target type	Effect on CMA	Effects on diseases
FDW028	FUT8	upstream molecule that regulates CMA	enhanced	Inhibition of metastatic CRC
Aspirin	HBXIP	upstream molecule that regulates CMA	enhanced	Reducing TAM resistance of breast cancer
Polyphyllin D	Hsc70 and LAMP2A	components of CMA	inhibited	Enhancing the sensitivity of NSCLC to cisplatin
PI3K/mTOR inhibitors	G6PD and Hsc70	substrate and component of CMA	enhanced	Attenuating radioresistance of SCLC
SAP	PD-L1 and Hsc70	substrate and component of CMA	enhanced	Restoring the antitumor immune response in metastatic melanoma
Romidepsin	HDAC2 and TPD52	upstream molecules that regulate CMA	inhibited	Preventing the tumor growth of PCa
AR7	LAMP2A	component of CMA	enhanced	Alleviating the symptoms of PD
β-asarone	MEF2D and TH	upstream molecules that regulate CMA	enhanced	Preventing brain damage in PD
Lactulose	N.A.	N.A.	enhanced	Reducing neuroinflammation and promoting the synaptic expression in AD
Trehalose	N.A.	N.A.	enhanced	Reducing neuroinflammation and promoting the synaptic expression in AD
NAC	N.A.	N.A.	enhanced	Alleviating NSAID-induced steatosis and hepatotoxicity
Baicalin	N.A.	N.A.	enhanced	Alleviating NSAID-induced steatosis and hepatotoxicity
QX77	ACSL4	substrate	enhanced	Preventing the generation of harmful lipid substances in diabetic retinopathy
Anisomycin	eEF1A1	substrate	enhanced	Inhibiting the replication of CVB in CVB infection
Pinacidil	CRT	substrate	inhibited	Attenuating myocardial I-R injury
Vitamin E	N.A.	N.A.	inhibited	Relieving status epilepticus
